# Dermoscopic and reflectance confocal microscopy findings in palisaded encapsulated neuroma (PEN): report of two histologically confirmed cases

**DOI:** 10.1093/omcr/omae003

**Published:** 2024-02-16

**Authors:** Hailey Konisky, Hubert Huho, Aashka Suvarnakar, Sharma Krishna, Albert Huho

**Affiliations:** Albert Einstein College of Medicine, Bronx, NY, USA; Upstate Dermatology, Clinical and MOH’s Services, Castleton-on-Hudson, NY, USA; Georgetown University, Washington, DC, USA; Georgetown University, Washington, DC, USA; Upstate Dermatology, Clinical and MOH’s Services, Castleton-on-Hudson, NY, USA

**Keywords:** palisaded encapsulated neuroma (PEN), reflectance confocal microscopy, dermoscopy, basal cell carcinoma

## Abstract

These medical case reports discuss the reflectance confocal microscopy (RCM) features of palisaded encapsulated neuromas (PEN), a relatively uncommon benign neural tumor that predominantly manifests in middle-aged females, most notably on the facial cheek. These reports feature two middle-aged females who both presented with concern for progressively enlarging cheek papules. Dermoscopy revealed the presence of symmetrical, dome-shaped papules with arborizing blood vessels that were not in sharp focus. RCM images revealed a honeycomb pattern, replete with follicular openings and slight papillary dermal fibrosis. Horizontally oriented blood vessels and vellus hair follicles, as well as prominent vellus hair bulbs were noted. The findings were initially perplexing and collectively raised the possibility of a skin adnexal neoplasm. However, subsequent biopsy confirmed a diagnosis of PEN in both cases. In retrospect, the neoplastic cells were beyond the imaging depths of RCM but the observed changes, including alterations in hair follicles on confocal microscopy, were likely due to pressure from the underlying tumor. Consideration of these features aids in diagnosing this unique yet benign neoplasm.

## INTRODUCTION

Palisaded encapsulated neuroma (PEN), also known as solitary circumscribed neuroma, is a rare and benign neural tumor characterized by the excessive growth of Schwann cells and axons. It can clinically be confused with conditions like basal cell carcinoma, intradermal nevi, cutaneous schwannomas, or other neuromas. Typically, PEN appears as small (ranging from 2 to 6 mm) skin-colored papules or nodules that are firm and rubbery to the touch. These growths are usually asymptomatic and solitary, often found on the faces of middle-aged women, especially on the cheeks [1]. As they gradually increase in size, patient concerns about potential neoplasms may arise.

One distinguishing feature of PENs is that they are not associated with any systemic diseases, unlike neurofibromas. The primary treatment for PEN is surgical excision, which is typically curative with a low risk of recurrence. However, for patients who prefer a non-surgical approach, conservative management can be considered. Since basal cell carcinoma is often a consideration during diagnosis, it can be clinically useful to identify features suggesting benignity using reflectance confocal microscopy (RCM) to avoid unnecessary aggressive excisions and scarring. However, information about RCM and dermoscopic features of PEN is limited, with very few case reports described in the English literature [2].

## REPORT OF CASES

**Figure 1:**
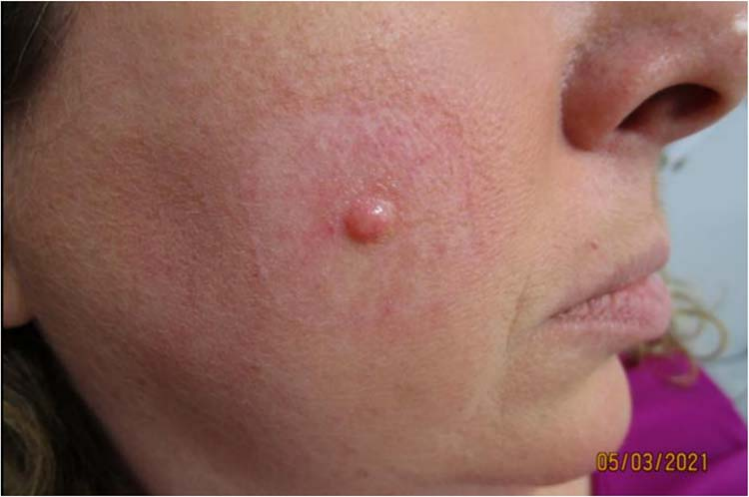
Case 1—50 year old female with small, solitary, firm, rubbery, skin-colored papule on right cheek.

A 50-year-old woman presented to our outpatient clinic (Upstate Dermatology, New York, USA) with a gradually enlarging, painless papule on her right cheek that had been progressively growing for 3 years (Fig. 1). She expressed concerns about the possibility of skin cancer, given her immediate family history of basal cell carcinoma (BCC). The second case involved a 56-year-old woman who presented with a slowly growing nodule on her left cheek, which had been a clinical concern for the past 5 years (Fig. 4). This patient had previously undergone successful Mohs surgery for BCC on her nose 6 years prior. Both cases exhibited clinical features that warranted an evaluation for potential skin cancer, due to either personal or family history. Neither of the cases had accompanying history of hearing defect or other neurological disorders. There were no family members affected by similar disorders and cutaneous examination of hair, nails, and mucous membranes were negative for disorders associated with phacomatoses.

### Dermoscopy

Upon non-polarized contact dermoscopy of both lesions we observed symmetrical, skin-colored, dome-shaped papules with arborizing vascular patterns, which were slightly more pronounced at the periphery but were not in sharp focus (Fig. 5). In the second case, a few vessels appeared more sharply focused. The differential diagnosis included nodular basal cell carcinoma, cutaneous neuroma, or an intradermal nevus.

### Reflectance confocal microscopy

**Figure 2:**
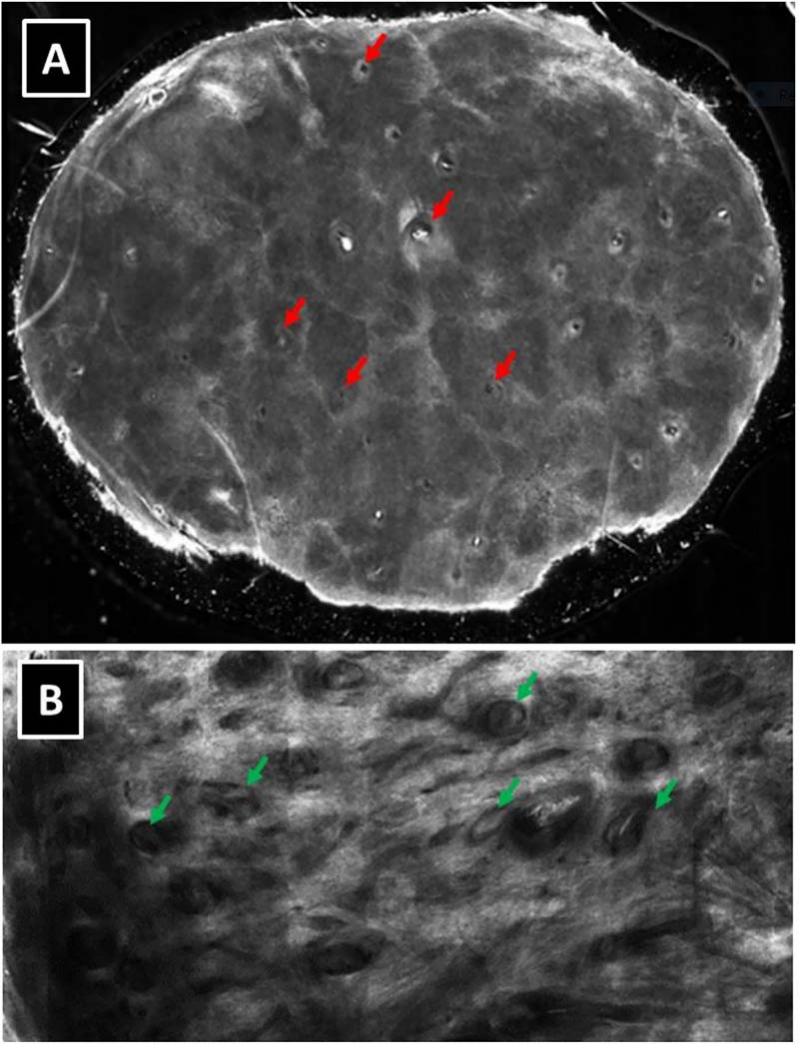
(Case 1) (**A**) RCM- note evenly distributed hair follicles (arrows) throughout the lesion, (**B**) RCM- deeper blocks revealed horizontally oriented hair follicles and Hair bulbs (arrows).

Reflectance confocal microscopy (RCM) examination did not reveal features indicative of basal cell carcinoma in either case. Instead, we identified a honeycombed pattern and multiple evenly distributed small follicular openings, which were faintly visible but not clearly appreciated through dermoscopy alone. Deeper tissue examination revealed horizontally oriented hair follicles ending in visible vellus hair bulbs (Figs 2 and 6). These findings raised suspicion of a skin adnexal neoplasm. The dermal-epidermal junction appeared intact, and there was moderate-to-marked papillary dermal fibrosis. Biopsies were performed in both cases. In the second case, the lesion was followed longitudinally for 6 months, during which RCM and dermoscopy showed a slight increase in size.

**Figure 3:**
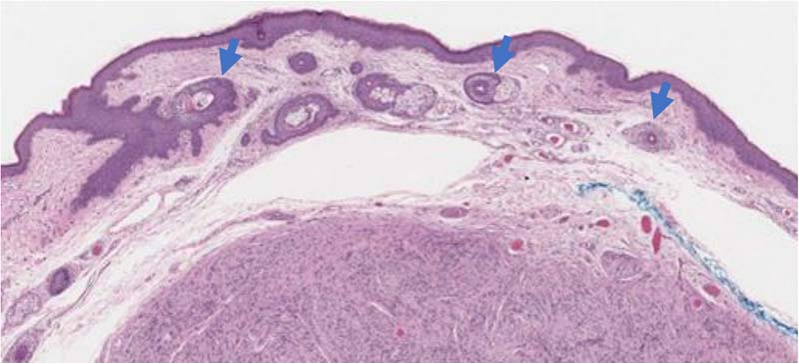
Case 1—Histology sections (40×, Hematoxylin and eosin stain) illustrating transections of horizontal hair follicles above the tumor (arrows).

**Figure 4:**
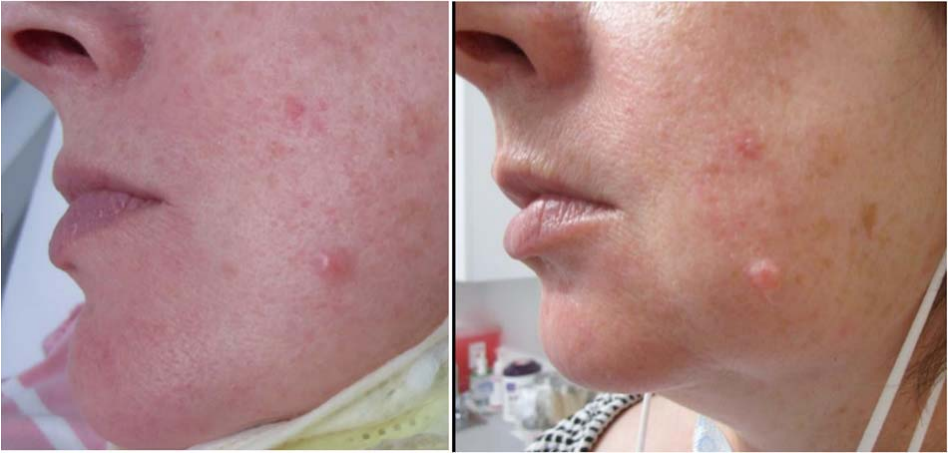
(CASE 2) A 56-year-old female, demonstrating a lesion on the left mandible, that has exhibited growth over the last 6 months. Observe the subtle enlargement of the lesion in the initial images (left side) compared to the follow-up images 6 months later (right side).

**Figure 5:**
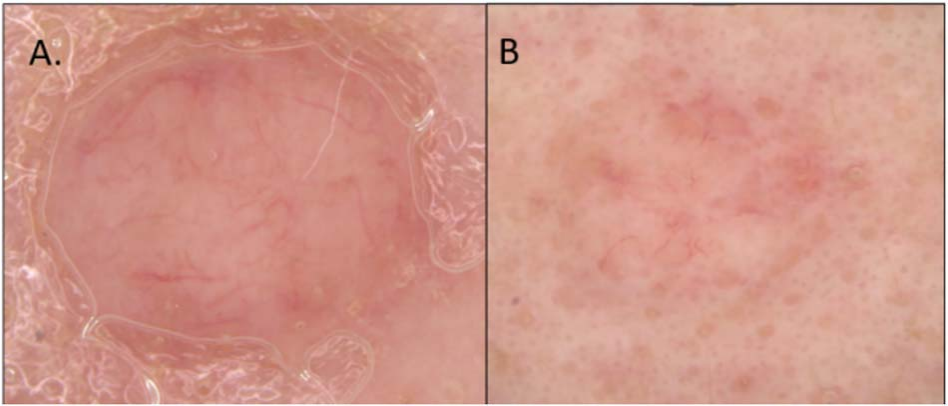
Dermoscopic images showing symmetric non-pigmented lesions with a vague arborizing vascular pattern. Note the vessels do not seem to be in sharp focus. Dermoscopy images from Case 1 (**A**) and Case 2 (**B**).

**Figure 6:**
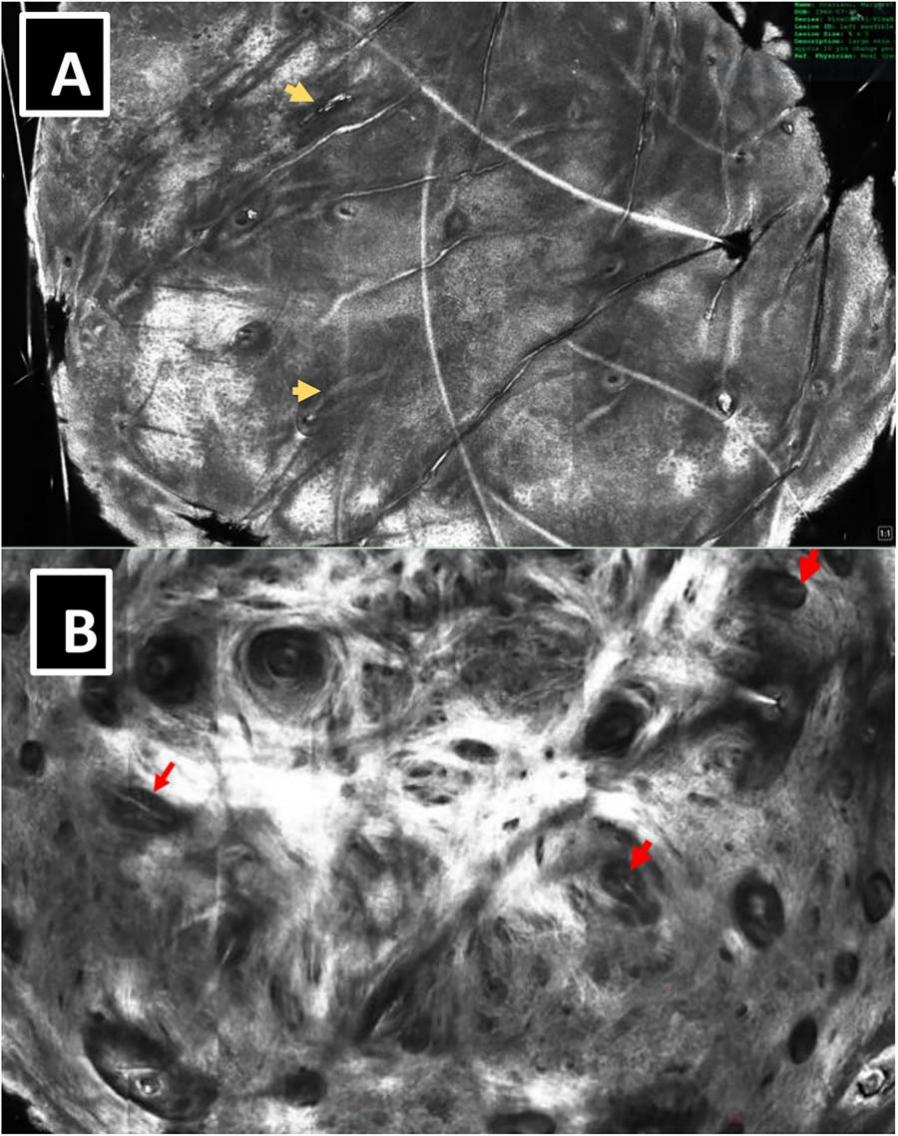
Case 2—(**A**) RCM- noted horizontally oriented hair follicles (arrows) throughout the lesion. (**B**) RCM- deeper blocks revealed prominent hair bulbs (arrows).

### Histology

Histological examination of both cases revealed well-circumscribed tumors located in the superficial dermis, composed of small, spindled cells without atypia or mitotic activity (Fig. 3). The encapsulated tumor was separated from the epidermis by a capsule and a grenz zone-like area. Correlating RCM findings with histology drew attention to the presence of multiple tilted hair follicles extending from the epidermis into the superficial papillary dermis. It’s noteworthy that the pseudo-encapsulated tumor cells were located too deep for RCM imaging (>150 μm) and were not captured in any of the RCM images obtained.

The only definitive treatment option available for PEN is surgical excision [3]. Receiving a diagnosis through biopsy or RCM/dermoscopy is reassuring because it confirms a benign condition without any connections to other syndromes. While neurofibromas have associations with neurofibromatosis 1 and underlying neurofibrosarcoma, and mucosal neuromas are linked with tumors of multiple endocrine neoplasia II, PEN is not related to any additional neoplasms. Once a definitive diagnosis is established, patients can find reassurance in the fact that excision is curative, with a low likelihood of recurrence. This eliminates the need for unnecessary further testing and, more importantly, dispels concerns about related systemic disease or malignancy.

## DISCUSSION

These two cases underscore the importance of recognizing specific dermoscopic and RCM findings associated with PEN. Initially, the dermoscopic appearance and vascular patterns raised concerns of BCC. However, the presence of evenly distributed follicular openings suggested a different diagnosis, as these are an uncommon feature in BCC cases, which usually displace hair follicles [4, 5]. In contrast, neuromas and dermal nevi typically display vertically oriented hair follicles within dermal lesions and rarely exhibit multiple visible hair bulbs.

The authors propose that the presence of multiple follicular openings and horizontally displaced vellus hairs with visible hair bulbs on the faces or cheeks of middle-aged females should prompt consideration of a PEN diagnosis. Although the individual spindle cells within these tumors were not visible within the imaging depth of RCM, the combination of clinical, dermoscopic, and RCM findings should raise suspicion of a benign palisaded encapsulated neuroma.

## CONCLUSION

The presence of a skin-colored, dome-shaped papule on the face accompanied by arborizing vascular patterns and the presence of horizontally displaced follicular units with visible hair bulbs could indicate a potential diagnosis of PEN. These 2 cases emphasize the presence of horizontally oriented hair follicles and multiple visible vellus hair bulbs as significant features suggestive of palisaded encapsulated neuromas. In summary, the findings in these cases underscore this correlation between RCM, dermoscopy, and histology for diagnosing PEN.
